# Bivariate Genome-Wide Association Analysis of the Growth and Intake Components of Feed Efficiency

**DOI:** 10.1371/journal.pone.0078530

**Published:** 2013-10-29

**Authors:** Nick V. L. Serão, Dianelys González-Peña, Jonathan E. Beever, Germán A. Bollero, Bruce R. Southey, Daniel B. Faulkner, Sandra L. Rodriguez-Zas

**Affiliations:** 1 Department of Animal Sciences, University of Illinois at Urbana-Champaign, Urbana, Illinois, United States of America; 2 Department of Crop Sciences, University of Illinois at Urbana-Champaign, Urbana, Illinois, United States of America; 3 Department of Statistics, University of Illinois at Urbana-Champaign, Champaign, Illinois, United States of America; 4 Institute for Genomic Biology, University of Illinois at Urbana-Champaign, Urbana, Illinois, United States of America; Wageningen UR Livestock Research, The Netherlands

## Abstract

Single nucleotide polymorphisms (SNPs) associated with average daily gain (ADG) and dry matter intake (DMI), two major components of feed efficiency in cattle, were identified in a genome-wide association study (GWAS). Uni- and multi-SNP models were used to describe feed efficiency in a training data set and the results were confirmed in a validation data set. Results from the univariate and bivariate analyses of ADG and DMI, adjusted by the feedlot beef steer maintenance requirements, were compared. The bivariate uni-SNP analysis identified (P-value <0.0001) 11 SNPs, meanwhile the univariate analyses of ADG and DMI identified 8 and 9 SNPs, respectively. Among the six SNPs confirmed in the validation data set, five SNPs were mapped to *KDELC2*, *PHOX2A*, and *TMEM40*. Findings from the uni-SNP models were used to develop highly accurate predictive multi-SNP models in the training data set. Despite the substantially smaller size of the validation data set, the training multi-SNP models had slightly lower predictive ability when applied to the validation data set. Six Gene Ontology molecular functions related to ion transport activity were enriched (P-value <0.001) among the genes associated with the detected SNPs. The findings from this study demonstrate the complementary value of the uni- and multi-SNP models, and univariate and bivariate GWAS analyses. The identified SNPs can be used for genome-enabled improvement of feed efficiency in feedlot beef cattle, and can aid in the design of empirical studies to further confirm the associations.

## Introduction

Optimization of feed efficiency in livestock production demands the consideration of the system inputs and outputs. In feedlot beef cattle enterprises, feed consumption dominates the input (and costs) and weight gain dominates the output (and return). Average daily gain (ADG) per animal, computed as the difference between final and initial trial weight divided by the number of days in the trial, is a frequently used indicator of weight gain. The cost of feed represents 62% to 84% of the total costs in a beef cattle production unit [1]. Dry matter intake (DMI) per day and animal is a frequently used indicator of feed consumption. In addition, 70 to 75% of the total energy feed intake is spent on maintenance functions (e.g. body temperature, digestion) in beef cattle [2]. Metabolic body weight (MBW) per animal, computed as BW^0.73^, is an accepted indicator of maintenance requirements.

Genomic improvement of feed efficiency in beef cattle relies on the identification of genomic variants (single nucleotide polymorphisms or SNPs) associated with feed efficiency components. A genome-wide association study (GWAS) can be used to identify SNPs to be included in genome-enabled selection decisions. The study of feed efficiency requires the consideration of output (ADG) and input (DMI) indicators, adjusted for maintenance requirements (MBW).

The majority of the SNPs reported to be associated with feed efficiency were identified from the analysis of each component (ADG or DMI) separately, or functions thereof such as residual feed intake and residual average daily gain [3]–[5]. On one hand, the analysis of feed efficiency components separately may fail to exploit the covariation between the components and consequently loose statistical precision to detect SNPs. On the other hand, the feed efficiency functions adjust either component by the other, thus imposing the selection of one component as the response and the assumption that the other component is an explanatory variable measured without error. Furthermore, the analysis of these functions fails to consider the uncertainty of the adjusted values. Bivariate analysis can augment the statistical precision to detect SNPs associated with both feed efficiency components. This gain stems from the consideration of covariation between the components that can augment the SNP signal relative to the noise or error [6]–[12]. No bivariate GWAS of feed efficiency components in beef cattle has been reported. The objectives of this study were: 1) to identify and characterize SNPs associated with feed efficiency components in a feedlot beef cattle population using bivariate analysis; 2) to compare the results from bivariate and univariate analyses; 3) to evaluate the results from uni- and multi-SNP models identified in a training data set on a validation data set; and 4) to enhance the interpretation of the results using functional genomic analyses and network visualization. Single nucleotide polymorphisms that exhibited favorable associations with both feed efficiency components, or that exhibited a favorable association with either component while minimizing a disfavorable trend on the other component were highlighted. These SNPs are well-suited for genome-enabled selection programs to improve feed efficiency and for follow-up empirical confirmation.

## Materials and Methods

### Ethics statement

All procedures were conducted following the guidelines recommended in the Guide for the Care and Use of Agricultural Animals in Agricultural Research and Teaching [13] with the approval of the University of Illinois Institutional Animal Care and Use Committee. The respective owners of the animals granted permission for their use in this study.

### Beef cattle steers studied

Measurements were collected from 1,321 feedlot steers from five ranches in Montana between 2005 and 2008. The combination of ranch, harvest group, and harvest year resulted on 27 contemporary groups (CGs). Pedigree and breed information from 3,331 animals [14] were used to define the breed composition of each steer and to infer the relationship matrix. Steers pertain to one of five breed compositions: purebred Angus (AN), 3/4 Angus (3/4AN), crossbred Angus and Simmental (ANSM), 3/4 Simmental (3/4SM), or purebred Simmental (SM). The trial lasted an average (± standard deviation) of 165 (±16) days. Each steer received one of the twelve diets [15]. The diets were further grouped into five dietary treatments according to the main ingredient, total net energy, and non-degradable fiber (Table 1, [15]).

**Table 1:** Description of the diets received by the beef cattle steers analysed.

	Diets				
Item	A	B	C	D	E
TNE, Mcal/lb	1.40	1.15	1.15	1.15	1.09
NDF, %	18.5	39.2	41.5	40.1	45.1
DM, %	66.7	63	65	54	49
CP, %	13.9	18.8	14.4	17.7	21.4
ADF, %	7.8	21.9	23.6	22.8	25.6
TDN, %	75.7	67.5	68	68	66
Main ingredients	Dry-rolled corn and stored wet distiller grain	Distiller grains with solubles and fresh wet corn gluten feed	Dry-rolled corn and corn gluten feed	Fresh wet distiller grains and wet corn gluten feed	Stored wet distiller grains and hay

TNE, Total net energy; NDF, Non-degradable fiber; DM, Dry matter; CP, Crude protein; ADF, Acid detergent fiber; TDN, Total digestible nutrient.

### Measurements

Two feed efficiency components, ADG and DMI, were analyzed. Individual steer ADG (kg) was the difference between adjusted final weight (FW) and initial weight (IW) in the trial divided by days in the trial. The FW was estimated by dividing the individual hot carcass weight by the average dressing percentage of the harvest group. Individual daily DMI (kg) was measured using the GrowSafe automated feeding system (GrowSafe Systems Ltd., Airdrie, Alberta, Canada). Individual MBW was calculated using the estimated BW mid-trial. The age of the steer at mid-trial (mA; days) was also recorded. The average (± standard deviation) ADG, daily DMI, IW, FW, MBW, and mA were: 1.61±0.24 kg, 10.48±1.42 kg, 310.10±40.08 kg, 597.50±48.43 kg^0.73^, 366.40±40.12 kg, and 332.58±29.32 days, respectively.

### Genotyping and quality control

The DNA was extracted from blood samples using the salting out method [16]. The SNP genotypes were obtained from Illumina® BovineSNP50 BeadChips v1 and v2 platforms (Illumina Inc., San Diego, CA) that include 54,001 and 54,609 SNPs, respectively. Quality control was performed in two steps on the 52,340 SNPs present in both platforms. In the first quality control step, SNPs not assigned to chromosomes, according to the Bos_taurus_UMD_3.1 assembly [17], and having a GenCall score <0.2 (suggesting unreliable genotype [18]) were filtered. From this step, 519 and 16 SNPs were excluded. The software PLINK v.1.07 [19] was used to perform the second quality control step. In this step, SNPs and steers were removed when not meeting either one of the following criteria: missing steer per SNP <20%) [20]; Hardy-Weinberg equilibrium test P-value >0.00001 [21]; missing SNP per steer <10% [22]; and minor allele frequency >5% [22]. After the second quality control step, 264 SNPs, 1,202 SNPs, 9 steers, and 9,811 SNPs were not considered for further analysis applying the previous criteria, respectively. The final data set included 1,312 steers, 40,528 SNPs, and a total genotyping rate of 99.55%.

### Statistical analyses

#### Uni- SNP model, univariate and multivariate analyses

Univariate (ADG or DMI) and bivariate (ADG and DMI) analyses of uni-SNP mixed-effect models were used to detect SNPs associated with feed efficiency. The uni-SNP model for the univariate analysis (Equation 1; Eq.1) was: 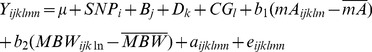
(1)where *Y_ijklmn_* denoted the observed ADG or DMI, *μ* denoted the overall mean, *SNP_i_* denoted the fixed effect of an individual SNP genotype, *B_j_* denoted the fixed effect of breed (5 levels), *D_k_* denoted the fixed effect of diet (5 levels, Table 1), *CG_l_* denoted the random contemporary group effect (27 levels) that has a Normal distribution (0, 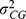), *b_1_* denoted the fixed effect regression coefficient for the covariate *mA*, *b_2_* denoted the fixed effect regression coefficient for the covariate *MBW*, *a_ijklmn_* denoted the random animal polygenic effect that has a Normal distribution (0, 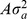) where *A* denoted the additive relationship matrix, and *e_ijklmn_* denoted the random normal distributed error (0, 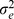). The corresponding bivariate analysis (Eq. 2) was: 
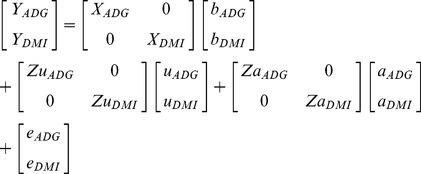
(2)where *Y_ADG_* and *Y_DMI_* denoted the vectors of observed ADG and DMI, respectively; *X_ADG_* and *X_DMI_* denoted the incidence matrices for the fixed effects for ADG and DMI, respectively; *b_ADG_* and *b_DMI_* denoted the vectors of solutions associated with *X_ADG_* and *X_DMI_*, respectively; *Zu_ADG_* and *Zu_DMI_* denoted the incidence matrices for the random contemporary groups for ADG and DMI, respectively; *u_ADG_* and *u_DMI_* denoted the vectors of solutions associated with *Zu_ADG_* and *Zu_DMI_*; respectively; *Za_ADG_* and *Za_DMI_* denoted the incidence matrices for the random animal polygenic effects for ADG and DMI, respectively; *a_ADG_* and *a_DMI_* denoted the vectors of solutions associated with *Za_ADG_* and *Za_DMI_*, respectively; and *e_ADG_* and *e_DMI_* denoted the vectors of random errors associated with *Y_ADG_* and *Y_DMI_*, respectively; assuming random effects distributed as multivariate Normal that had mean equal to zero and covariance matrix: 
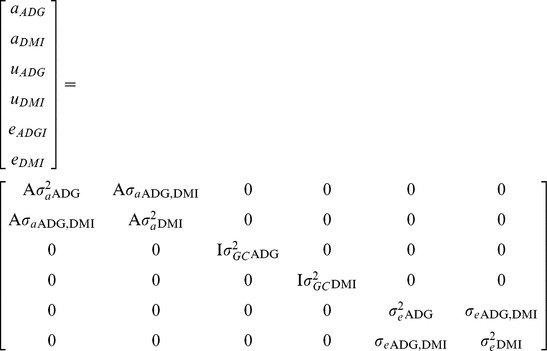
where 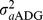 and 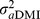 denoted the random animal polygenic variance for ADG and DMI, respectively; 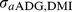 denoted the random animal polygenic covariance between ADG and DMI; 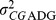 and 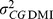 denoted the random contemporary group variance for ADG and DMI, respectively; *I* denoted the identity matrix; 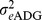 and 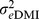 denoted the random error variance for ADG and DMI, respectively; and 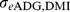 denoted the random error covariance between ADG and DMI. The models used in the univariate and bivariate analyses included the same explanatory variables.

The GWAS was implemented using Qxpak v.5.05 [23] and SNPs exhibiting associations with the feed efficiency components at an unadjusted P-value <0.0001 were deemed significant. The additive and dominance effects were estimated for SNPs on autosomal chromosomes, and the additive effect was estimated for SNPs located on chromosome X. The additive effect estimate was computed relative to the less frequent (minor) allele among the steers studied. The additive effect estimate was defined as the change on the feed efficiency component per additional minor allele in the SNP genotype. The dominance effect estimate was defined as the difference on the feed efficiency component between the heterozygous and homozygous steers. Model assumptions including independence of residuals, homogeneity of variance, and normality were evaluated.

#### Multi-SNP model selection

A multi-SNP model was developed for the univariate and bivariate analyses. Starting with the SNPs detected at P-value <0.001 in the uni-SNP models and all other explanatory variables equal, a stepwise feature selection approach was used. The final multi-SNP model included the SNPs that entered (were added to the model) and stayed (were kept in the model after consideration of all other SNPs in the model) in the multi-SNP model at P-value <0.0001.

#### Training and validation of the uni- and multi-SNP results

The SNPs were first identified using complementary models and analyses on a training data sets. Subsequently, the findings were evaluated on a validation data set that included a separate group of steers. Training and validation data sets were generated from the records that passed the quality control based on sire family [4], [24], [25]. Steers were randomly assigned to either the training set (976 steers; 75%) or the validation set (336 steers; 25%; Table 2). Data partitioning ensured that each sire was represented in only one of the data sets to minimize potential confounding between SNP and individual associations [4], [24].

**Table 2:** Number (proportion) of steers by breed and diet in the training and validation data sets.

Training (*n* = 976)				Validation (*n* = 336)			
Breed		Diet		Breed		Diet	
AN	102 (0.10)	A	232 (0.24)	AN	35 (0.10)	A	83 (0.25)
3/4AN	115 (0.12)	B	300 (0.31)	3/4AN	67 (0.20)	B	88 (0.26)
ANSM	640 (0.66)	C	111 (0.11)	ANSM	190 (0.57)	C	48 (0.14)
3/4SM	39 (0.04)	D	257 (0.26)	3/4SM	19 (0.06)	D	105 (0.31)
SM	80 (0.08)	E	76 (0.08)	SM	25 (0.07)	E	25 (0.07)

The SNPs detected (P-value <0.0001) in the training data set using the uni-SNP model and univariate and bivariate analyses were validated at P-value <0.05 [24]. The trend (sign) of the genetic estimates was also compared between the training and validation data sets. For the multi-SNP models, the SNPs were validated based on the change in the model prediction accuracy, termed model adequacy (MA), between training and validation data sets. For the univariate analyses of ADG and DMI, the square root of the mean square error (RMSE) was used to indicate the difference between the observed and predicted values and thereof, model inadequacy. The change in MA for the univariate analyses of ADG and DMI (Eq. 3) was: 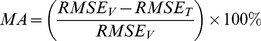
(3)where *RMSE_V_* is the RMSE from the validation data set and *RSME_T_* is the RMSE from the training data set. For the bivariate analysis, model inadequacy was the average of three root mean (co)variance terms: RMSE for ADG, RMSE for DMI, and the root means square covariance (RMSC) between ADG and DMI. The change in MA was computed as for the univariate analysis.

#### Linkage disequilibrium

Some of the SNPs detected could be an artifact of the dependency between SNPs that exhibit high linkage disequilibrium (LD). This situation is the result of the average probe spacing (49.4 kb) of the platform [26] and the large number of SNPs tested. Statistical dependencies between significant SNP pairs located less than 500 kb apart that could suggest LD was assessed using the standard *r^2^* statistic [27] in PLINK. The LD extent in cattle is estimated to be 500 kb [28].

#### Genetic parameters

The genetic parameters of ADG and DMI were estimated to assess the potential amount of genetic variability that could be associated to SNPs. Heritability and genetic and phenotypic correlations between ADG and DMI were estimated using an animal model and univariate and bivariate analyses implemented in WOMBAT [29], [30]. The explanatory variables included in the animal models encompassed those described in Eq. 4.

#### Functional and gene network analyses

The detected SNPs were mapped to harboring or proximal (within 2 kb of the 5′ untranslated region or 0.5 kb of the 3′ untranslated region of a gene) genes in the Bos_taurus_UMD_3.1 assembly. The SNP mapping and gene information was obtained from the National Center for Biotechnology Information, SNP and Gene databases [31].

Functional analysis of the genes corresponding to the SNPs detected (P-value <0.01) in the bivariate analysis offered insights into the categories enriched among the genes. The consideration of genes from the bivariate analysis was motivated by the goal of identifying functional categories among genes that could have pleiotropic effects on both feed efficiency components. Genes farther upstream and downstream from the detected SNPs were not included in the functional analysis because the number of spurious (false positive) genes added to the functional analysis could have overwhelmed the fewer real (true positive) loci, potentially biasing the results. The enrichment of Gene Ontology (GO) FAT categories and KEGG pathways among the genes was investigated using Fisher's exact test in DAVID [32], [33]. The GO FAT categories are a subset of the broadest GO terms, filtered to minimize overshadowing of more specific terms due to repetition of more general categories. Functional annotation charts were considered significant at P-value <0.001 using the *Bos Taurus* genome as background.

Gene networks associated with feed efficiency were visualized using the genes affiliated to the enriched functional categories. The network was visualized using the BisoGenet plug-in Cytoscape [34], with default settings. Identified (or target) genes and intermediate connecting genes from the NCBI database genes were represented by nodes. The final pathway included target genes separated by at most two intermediate genes. Edges denoted known relationships between genes.

## Results and Discussion

### General results

The heritability estimates of ADG and DMI were 0.14 and 0.25, respectively. These heritability estimates confirmed the opportunity for genome-based improvement of these feed efficiency components. The phenotypic and genetic correlations between ADG and DMI were 0.52 and 0.18, respectively. These estimates were consistent or slightly lower than in previous reports [35], [36]. The positive genetic correlation supports the hypothesis that SNP alleles that have positive association with ADG and negative association with DMI could be identified and could assist with genome-based improvement feed efficiency in beef cattle.

A summary of the number of significant SNPs (and corresponding genes) detected by the univariate and bivariate analyses is presented in Table 3. Among the 28 SNPs detected (P-value <0.0001), 19 SNPs overlapped between the univariate and bivariate analyses. The bivariate analysis detected the highest number of SNPs (11 SNPs) followed by the univariate analyses of DMI (9 SNPs) and ADG (8 SNPs). Similar associations between SNPs and ADG or DMI have been previously reported [37], [24]. The partial overlap of SNPs confirmed the complementary information offered by the univariate and bivariate analyses. For certain SNPs, the bivariate analyses could gain precision through the consideration of covariation between ADG and DMI relative to univariate analyses. For other SNPs, univariate analyses benefited from lower noise of each trait studied separately, relative to the bivariate analysis. Associations with SNPs were identified on 10 chromosomes (Tables 4 and 5): BTA 2, 4, 6, 13, 14, 15, 17, 22, 23, and 26. The highest number of SNPs was identified on BTA15 (10 SNPs) followed by BTA13 (3 SNPs) and BTA17 (3 SNPs).

**Table 3:** Number of detected SNPs and corresponding genes across P-value thresholds by analysis.

	P-value <0.0001		P-value <0.001		P-value <0.01	
Analysis	SNPs	Genes	SNPs	Genes	SNPs	Genes
ADG	8	6	53	21	413	153
DMI	9	3	58	18	560	206
Bivariate	11	5	84	34	587	236
Total^1^	19	9	146	51	1126	419

1 Unique SNPs and genes.

The potential relationship between feed efficiency and the genes harboring or in the proximity of the detected SNPs was investigated. The connection between the feed efficiency components and genes was based on gene annotation information available at the National Center for Biotechnology Information, Gene database [31]. This information was complemented with literature review where relevant.

### Univariate uni-SNP analysis of ADG

Previous studies reported genomic regions associated with ADG on BTA 2, 4–7, 9, 11, 14–20, 22, 23, 26, and 28 [38]–[52]. The SNPs associated with ADG in the present study (Table 4) were mapped to chromosomes previously linked to ADG, with the exception of rs41629972 located on BTA13. This SNP is located approximately 33 kb upstream Kruppel-like factor 6 (*KLF6*) and is within the 500 kb LD span reported in cattle [28], [29]. The zinc finger protein encoded by this gene has been associated with cell proliferation, differentiation, signal transduction, and cell death [31], [53], [54]. *KLF6* regulates genes in the transforming growth factors β signaling pathway [55]. Transforming growth factors β superfamily members affect both muscle development and postnatal skeletal muscle mass [31], [56], [57]. Every additional T allele was associated with 0.04 kg higher ADG relative to the C allele. Also associated with ADG but not mapped to a gene, rs41565199 was mapped on BTA14 within LD reach (464 kb downstream) of zinc finger and homeobox 2 (*ZHX2*). This QTL region has been linked to ADG in Japanese Black (Wagyu) cattle [47]. Homozygous CC steers exhibited the highest ADG.

**Table 4:** P-values and estimates^1^ of the SNPs detected^2^ by the uni-SNP univariate analysis of feed efficiency.

								P-value^4^	
Trait	SNP	BTA	Allele	Gene Symbol	Gene Name	Additive^3^	Dominance^3^	T	V
ADG	rs109934193	2	C*/T	*NCKAP5*	NCK-associated protein 5	0.03±0.01	0.03±0.01	1.91E-05	9.04E-01
	rs110787048	4	A*/G	*DPP6*	Dipeptidyl-peptidase 6	0.03±0.01	0.06±0.01	5.40E-05	8.31E-01
	rs41629972	13	C/T*			0.04±0.01	0.01±0.01	7.22E-05	3.20E-02
	rs41565199	14	C*/T			0.03±0.01	−0.04±0.01	5.16E-05	9.42E-01
	rs41620774	15	A/C*	*ELMOD1*	ELMO/CED-12 domain containing 1	−0.15±0.04	−0.18±0.04	2.99E-05	3.01E-01
	rs108964818	15	C/T*	*KDELC2*	KDEL (Lys-Asp-Glu-Leu) containing 2	−0.40±0.09	−0.39±0.09	6.52E-06	3.60E-02
	rs41768978	15	A*/C	*PHOX2A*	Paired-like homeobox 2a	0.01±0.01	0.06±0.01	1.18E-05	1.03E-01
	rs42342964	23	G*/T	*PAK1IP1*	PAK1 interacting protein 1	0.01±0.01	0.06±0.01	9.04E-05	4.63E-01
DMI	rs41663978	6	A*/C			−0.22±0.05	−0.01±0.06	6.33E-05	8.71E-02
	rs41588990	6	A*/G	*CNOT6L*	CCR4-NOT transcription complex, subunit 6-like	0.01±0.06	0.29±0.07	3.55E-05	1.37E-01
	rs41632270	13	G/T*			−0.13±0.09	−0.40±0.10	9.00E-05	9.41E-01
	rs42128656	15	A*/G			−0.19±0.05	−0.10±0.06	2.20E-05	9.52E-01
	rs43291568	15	A*/G	*CLMP*	CXADR-like membrane protein	−0.25±0.05	−0.04±0.06	3.42E-06	8.44E-01
	rs43291603	15	C/T*	*CLMP*	CXADR-like membrane protein	−0.29±0.09	0.14±0.10	4.18E-05	7.44E-01
	rs111010038	17	A*/C			0.35±0.18	−0.50±0.19	2.41E-05	8.95E-01
	rs108942504	22	A/G*	*TMEM40*	Transmembrane protein 40	0.31±0.09	−0.02±0.11	2.26E-05	1.69E-02
	rs41624569	26	A*/G			−0.04±0.05	−0.27±0.06	1.94E-05	7.41E-01

1 Additive estimate relative to the minor allele;

2 P-value <0.0001;

3 Estimate ± standard error;

4 T, training data set; V, validation data set;

*Minor allele.

The remaining six SNPs associated with ADG were mapped to known genes. Steers heterozygous for rs109934193 on BTA2and rs110787048 on BTA4, had higher ADG than the average homozygous steer. These SNPs were mapped to NCK-associated protein 5 (*NCKAP5*) and dipeptidyl-peptidase 6 (*DPP6*), respectively. The three SNPs on BTA15 associated with ADG were: rs41620774 mapped to engulfment and cell motility/CED-12 domain containing 1 (*ELMOD1*), rs108964818 mapped to Lys-Asp-Glu-Leu containing 2 (*KDELC2*), and rs41768978 mapped to paired-like homeobox 2a (*PHOX2A*). Both rs41620774 and rs108964818 were mapped less than 1 Mb apart, and for both SNPs, steers homozygous for the minor allele (CC and TT, respectively) had the lowest ADG. The protein encoded by *ELMOD1* has a GTPase-activator function on small G proteins of the arf family [58]. These proteins have a central role in the organization of the secretory and endocytic pathways [59]. Mapped 4 Mb from a QTL previously associated with ADG [45], steers heterozygous for rs41768978 exhibited 0.060 kg higher ADG than the average homozygous steer. This SNP was mapped to the intronic region of *PHOX2A*, a gene associated with respiratory rhythm (and thus biochemical energy) and autonomic nervous system development [31], [60]. Lastly rs42342964, mapped to the PAK1 interacting protein 1 (*PAK1IP1*) on BTA23, was associated with ADG. The p21-activated protein kinase-interacting protein 1 encoded by this gene has been associated with cell proliferation and signal transduction [31], [60].

### Univariate uni-SNP analysis of DMI

The SNPs associated with DMI are presented in Table 4. Previous reports indicate genomic regions associated with DMI on all chromosomes, except BTA 19, 27–29, and X [3], [45], [48], [50], [51], [61]–[63]. The SNPs detected in the present study were mapped to BTA previously reported, with the exception of rs108942504 and rs41624569 on BTA 22 and 26, respectively. In particular, rs41624569 was mapped within the 500 kb LD region of several genes including: ATPase family, AAA domain containing 1 (*ATAD1*), lipase, family member J (*LIPJ*), 3′-phosphoadenosine 5′-phosphosulfate synthase 2 (*PAPSS2*), phosphatase and tensin homolog (*PTEN*), and renalase, FAD-dependent amine oxidase (*RNLS*). These genes play roles in mechanisms related to energy expenditure, including *ATAD1* role on ATP catabolism [60], *LIPJ* role on lipid catabolism [60], and *PTEN* role on inositol phosphate metabolism [64]. Steers heterozygous for rs41624569 exhibited the lowest daily DMI, approximately 0.270 kg less than the average homozygous steer.

Four additional SNPs associated with DMI were not mapped to genes. Every additional C allele on rs41663978 was associated with lower daily DMI (−0.22±0.05 kg) relative to the A allele. This SNP was mapped on BTA6, approximately 5 Mb from a QTL previously associated with DMI [45]. In addition, rs41663978 is located within 500 kb from ADAM metallopeptidase with thrombospondin type 1 motif, 3 (*ADAMTS3*), group-specific component vitamin D binding protein (*DBP*), and neuropeptide FF receptor 2 (*NPFFR2*). These genes have been associated with protein processing [60], leanness [31], and obesity [31], respectively. Similarly, rs41632270 was mapped to a QTL region on BTA13 associated with DMI [63] and within 500 kb of several genes including kinesin family member 16B (*KIF16B*), N-acetylneuraminic acid phosphatase (*NANP*), otoraplin (*OTOR*), phosphoribosylaminoimidazole carboxylase pseudogene (*PAICSP*), and small nuclear ribonucleoprotein polypeptide B (*SNRPB2*). In particular, *NANP* muschlparticipates on the amino sugar and nucleotide sugar metabolism [31]. Heterozygous steers for rs41632270 exhibited the lowest daily DMI (−0.40±0.10 kg) relative to the average homozygous steer.

Although rs42128656 was associated with DMI, this SNP was not mapped to a known gene. This SNP was 600 kb from other two SNPs (rs43291568 and rs43291603) on BTA15 also associated with DMI. This pair of SNPs were mapped within 27 kb of each other, however the pairwise LD among these SNPs was low (*r^2^* = 0.052). These two SNPs are in the intronic region of the coxsackie virus and adenovirus receptor-like membrane protein (*CLMP*). This gene encodes for a type I transmembrane protein of the CTX family and transmemrane proteins have activating or suppressing roles on cell growth [65].

Among the SNPs associated with DMI and mapped to known genes, rs108942504 was found on a gene on BTA22 that encodes a structural protein, the transmembrane protein 40 (*TMEM40*). Similarly, rs41588990 was mapped to the intronic region of CCR4-NOT transcription complex, subunit 6-like (*CNOT6*) on BTA6. This gene plays a role in the deadenylation of mRNAs in the cytoplasm and deadenylation has been associated with cell growth [66]. The results from the univariate GWAS offer a first glimpse of SNPs that could be used in genomic improvement of feed efficiency. However, univariate analyses may miss detecting SNPs because these analyses do not exploit the correlation between ADG and DMI. This situation could be especially detrimental in scenarios with limited data size, limited effect of the SNP or limited disequilibrium between the SNP and the QTL influencing the feed efficiency components. Bivariate uni- and multi-SNP analyses shed additional light on these SNPs.

### Bivariate uni-SNP analysis of ADG and DMI

The SNPs simultaneously associated with ADG and DMI (P-value <0.0001) were presented in Table 5. Many of these SNPs that have a pleiotrophic association with both feed efficiency components were also found in either univariate analysis. The SNPs that overlapped between the univariate and bivariate analyses were summarized in Table 6. Four of the eleven SNPs detected in the bivariate analysis were also detected in the univariate ADG analysis and other five SNPs were detected in univariate DMI analysis (Table 6). The two additional SNPs detected by the bivariate analysis but not detected by the univariate analyses were rs41722387 and rs110522962. These results emphasize the need to consider the results of multivariate and univariate GWAS to precisely detect and characterize SNPs associated with feed efficiency.

**Table 5:** P-values and estimates^1^ of the SNPs detected^2^ by the uni-SNP bivariate analysis of feed efficiency.

					ADG		DMI		P-value^4^	
SNP	BTA	Allele	Gene Symbol	Gene Name	Additive^3^	Dominance^3^	Additive^3^	Dominance^3^	T	V
rs109934193	2	C*/T	*NCKAP5*	NCK-associated protein 5	0.04±0.01	0.03±0.01	0.06±0.05	0.15±0.06	6.84E-05	9.60E-01
rs41629972	13	C/T*			0.04±0.01	0.01±0.01	0.20±0.05	0.02±0.06	6.82E-05	1.07E-01
rs41722387	14	G*/T			−0.04±0.01	−0.01±0.01	−0.04±0.05	0.14±0.06	9.27E-05	1.69E-01
rs108964818	15	C/T*	*KDELC2*	KDEL (Lys-Asp-Glu-Leu) containing 2	−0.40±0.09	−0.39±0.09	−0.02±0.01	−0.05±0.48	3.71E-07	4.05E-02
rs42128656	15	A*/G			−0.03±0.01	0.03±0.01	−0.19±0.05	−0.10±0.05	2.07E-05	2.85E-01
rs43291568	15	A*/G	*CLMP*	CXADR-like membrane protein	−0.02±0.01	−0.01±0.01	−0.25±0.05	−0.05±0.06	5.85E-05	5.62E-01
rs41768978	15	A*/C	*PHOX2A*	Paired-like homeobox 2a	0.01±0.01	0.06±0.01	0.13±0.07	0.11±0.08	1.90E-05	8.35E-03
rs111010038	17	A*/C			−0.13±0.04	0.12±0.05	0.34±0.16	−0.48±0.17	6.64E-06	3.66E-01
rs110522962	17	C/T*			0.08±0.03	−0.05±0.03	−0.47±0.18	0.30±0.20	5.89E-05	3.81E-02
rs108942504	22	A/G*	*TMEM40*	Transmembrane protein 40	−0.02±0.01	−0.04±0.02	0.37±0.10	−0.04±0.12	7.92E-05	9.32E-02
rs41624569	26	A*/G			−0.01±0.01	−0.01±0.01	−0.03±0.05	−0.24±0.06	5.88E-05	1.80E-01

1 Additive estimate relative to the minor allele;

2 P-value <0.0001;

3 Estimate ± standard error;

4 T, training data set; V, validation data set;

*Minor allele.

**Table 6:** SNPs detected^1^ bymultiple analyses using uni-SNP models.

SNP	BTA	Gene Symbol	Phenotype
rs109934193	2	*NCKAP5*	ADG, Bivariate
rs41629972	13	*-*	ADG, Bivariate
rs108964818	15	*KDELC2*	ADG, Bivariate
rs42128656	15	-	DMI, Bivariate
rs43291568	15	*CLMP*	DMI, Bivariate
rs41768978	15	*PHOX2A*	ADG, Bivariate
rs111010038	17	-	DMI, Bivariate
rs108942504	22	*TMEM40*	DMI, Bivariate
rs41624569	26	-	DMI, Bivariate

1 P-value <0.0001.

Among the SNPs uncovered by the uni-SNP bivariate analysis, rs41722387 was mapped approximately 450 kb from rs41565199, a SNP associated with ADG on BTA14 (Table 4). Despite being physically close, the LD between these SNPs was low (*r^2^* = 0.046). Furthermore, rs41565199 was mapped to pseudo metalloendopeptidase (*OMA1*) that inhibits growth and approximately 400 kb upstream from hyaluronan synthase 2 (*HAS2*) that mediates cellular growth [31]. Homozygous TT steers for rs41565199 exhibited the highest feed efficiency due to higher ADG and lower DMI relative to steers that had other genotypes. The other SNP detected solely by the bivariate analysis was rs110522962. Mapped on BTA17, this SNP was approximately 4 Mb from a QTL region previously associated with ADG [48]. Homozygous TT steers exhibited the highest ADG and lowest daily DMI relative to steers that had other genotypes.

Among the remainder nine SNPs detected by the bivariate uni-SNP analyses, rs42128656, rs111010038, and rs108942504 were also associated with DMI in the univariate analyses. Six additional SNPs were detected by the bivariate analyses and had the same signs for both feed efficiency components. Positive associations of the same allele with both feed efficiency components are not always undesirable because a significant increase in ADG could compensate for a less significant increase in DMI. For example, rs108964818 (that maps to *KDELC2*) had positive associations with ADG and DMI albeit a much higher additive estimate for ADG than DMI. The SNPs detected by the one univariate and the bivariate analyses (Table 6) were also detected at a less stringent threshold (P-value <0.01; data not shown), by the other univariate analysis.

### Univariate and multivariate multi-SNP analyses

The polygenic nature of ADG and DMI can be adequately described with a multi-SNP model. In turn, the multi-SNP function can be used to predict feed efficiency or in genome-enabled selection programs to improve feed efficiency. Findings from the un-SNP analyses were used to develop a multi-SNP predictive equation. A multi-SNP model was developed using stepwise selection and 53, 58, and 84 SNPs (P-value <0.001) identified by the uni-SNP univariate ADG, DMI and bivariate analyses. The final multi-SNP ADG, DMI, and the bivariate models included nine, eight, and seven SNPs, respectively (P-value <0.0001; Table 7). These SNPs encompassed 21 unique SNPs on 10 genes. Among these, 11 SNPs were detected by the uni-SNP analyses.

**Table 7:** SNPs selected^1^ for the multi-SNP models, corresponding gene, and model adequacy indicator.

				RMSE^2^						
Phenotype	SNP	BTA	Gene Symbol	T				V		MA^4^
ADG	rs108939474	2	-	0.1049				0.1187		11.68%
	rs109934193	2	*NCKAP5*							
	rs110787048	4	*DPP6*							
	rs42433916	7	-							
	rs109945988	11	-							
	rs109957444	14	*FAM135B*							
	rs42230512	14	*TATDN1*							
	rs108964818	15	*KDELC2*							
	rs41768978	15	*PHOX2A*							
DMI	rs41588990	6	*CNOT6L*	0.5242				0.5650		7.21%
	rs41577108	13	-							
	rs41629972	13	-							
	rs41632270	13	-							
	rs41577655	15	-							
	rs43291568	15	*CLMP*							
	rs110911295	20	-							
	rs41624569	26	-							
				**RMSE** 2						
				**ADG**		**DMI**		**RMSC** 3		
				**T**	**V**	**T**	**V**	**T**	**V**	**MA** 4
Bivariate	rs108964818	15	*KDELC2*	0.0802	0.0985	0.3700	0.4672	0.0949	0.1166	19.40%
	rs110522962	17	-							
	rs41624569	26	-							
	rs41600811	22	-							
	rs109709275	15	*GRAMD1B*							
	rs42459305	29	-							
	rs109945988	11	-							

1 P-value <0.0001;

2 Root mean square errors (RMSE) for the models using the training (T) and validation (V) data sets;

3 Root means square covariance (RMSC) for the bivariate model using the training (T) and validation (V) data sets;

4 MA, Model adequacy as defined in Eqs. 3 and 4, for the univariate and bivariate analyses, respectively.

The additional SNPs uncovered by the multi-SNP approach were mapped on BTA 2, 7, 11, 13, 20, 22, and 29. On BTA2, rs108939474 was associated with ADG and was within 500 kb of heparan sulfate 6-O-sulfotransferase 1 (*HS6ST1*), UDP-glucose glycoprotein glucosyltransferase 1 (*UGGT1*), and Sin3A-associated protein 130 kDa (*SAP130*). These genes are known for their roles on glycosaminoglycan biosynthesis pathway and cell growth [64], metabolism of protein [64], and histone H3 acetylation [60], respectively. Also associated with ADG, rs42433916 was mapped approximately 160 kb downstream from the zinc finger protein 608 (*ZNF608*) on BTA7, and rs109945988 was mapped 4 Mb from a QTL on BTA11 previously associated with ADG [48], and within 500 kb from the genes baculoviral IAP repeat containing 6 (*BIRC6*), RAS guanyl releasing protein 3 (calcium and DAG-regulated, *RASGRP3*), tetratricopeptide repeat domain 27 (*TTC27*), and latent transforming growth factor beta binding protein 1 (*LTBP1*) that is associated with cell growth [31].

Among the SNPs included in the multi-SNP univariate DMI analysis, rs41629972 was detected by the uni-SNP DMI analysis. Also on BTA13, rs41577108 was proximal to CUGBP Elav-like family member 2 (*CELF2*). This gene modulates the cellular apoptosis program [31] and also proximal to enoyl CoA hydratase domain containing 3 (*ECHDC3*), and USP6 N-terminal like (*USP6NL*). Two additional SNPs in the multi-SNP univariate DMI analysis were rs41577655 and rs110911295. Mapped to BTA15, rs41577655 was located less than 250 kb upstream of apoptosis inhibitor 5 (*API5*) and tetratricopeptide repeat domain 17 (*TTC17*) that was associated with growth. Mapped to BTA20, rs110911295 was located 500 kb upstream from the PAP associated domain containing 7 (*PAPD7*) and steroid-5-alpha-reductase, alpha polypeptide 1 (3-oxo-5 alpha-steroid delta 4-dehydrogenase alpha 1; *SRD5A1*), and downstream the mediator complex subunit 10 (*MED10*) and NOP2/Sun domain family, member 2 (*NSUN2*). Both, *SRD5A1* and *NSUN2* have been associated with abdominal subcutaneous and visceral fat [31].

The multi-SNP model selection for the bivariate analysis of ADG and DMI resulted in three SNPs not detected by the uni-SNP bivariate model. Among these, rs109945988 was associated in the multi-SNP ADG model, and rs41600811 and rs42459305 represent new associations. In particular, rs41600811 was mapped 100 kb downstream the cell adhesion molecule with homology to L1CAM (close homolog of L1; *CHL1*) on BTA22. The cell–cell adhesion function of this gene enables cells to assemble into organized tissues. The remaining SNP, rs42459305, was mapped to a gene dense region on BTA29 with several predicted loci and located less than 3 kb downstream from the olfactory receptor, family 8, subfamily G, member 5 (*OR8G5*) and could impact feed consumption.

### Validation

The SNPs detected on the training data set were evaluated on the validation data set. Findings from the uni-SNP models were confirmed at P-value <0.05 in the validation data set. This threshold was used for two reasons. First, this validation constitutes the second of the two-phase approach. The SNPs have already been detected in the training data set at a P-value <0.0001. Second, a limited number of SNPs required validation. The SNPs (and analyses) validated were: rs108942504 (univariate DMI); rs41629972 and rs108964818 (univariate ADG); and rs108964818, rs41768978, and rs110522962 (bivariate).

For the multi-SNP models, validation was assessed by the change in the MA between the validation and training data sets, relative to the validation set (Table 7). Overall, the MA in the small validation data set was comparable to that in the larger training data set used to detect the SNPs. This result confirmed that the SNPs detected have a high likelihood to be replicable in additional populations. For the multi-SNP univariate DMI (ADG) analysis, the RMSE only increased 7.21% (11.67%) despite the fact that the validation data set was 300% smaller than the training data set. The higher loss in MA for the bivariate multi-SNP analysis on the validation data set (19.4%) may be due the higher parameterization of the model and lower precision of each estimate relative to univariate analyses.

### Functional analyses and gene networks visualization

The 236 genes corresponding to the SNPs detected at P-value <0.01 by the uni-SNP bivariate analysis were considered for functional analysis. The P-value threshold was selected because of the high number of SNPs detected by the univariate analyses at P-value <0.01 that were also detected in the bivariate analysis.

Seven functional categories were enriched (P-value <0.001) among the genes corresponding to the detected SNPs (Table 8). The most significant categories included the GO molecular functions of cation channel activity and metal ion transmembrane transporter activity. Both categories encompassed 10 genes. Affiliated to these two GO categories was transient receptor potential channel 2 (*TRPC2*). This gene was reported to be associated with several behavioral responses [60] and could be related to consumption and energy maintenance requirements. This gene corresponded to rs41603221 that was detected (P-value  = 0.0006) in the bivariate analysis. Furthermore, this SNP was mapped 4 Mb from a QTL reported to be associate with ADG on BTA 15 [45].

**Table 8:** Enriched^1^ Gene Ontology molecular functions from SNPs detected by the bivariate uni-SNP analysis.

Gene Ontology term	Genes	P-value
cation channel activity	*CACNB2*, *FGF2*, *KCNH1*, *KCNH7*, *KCNH8*, *KCNIP4*, *KCNK9*, *KCNMA1*, *KCNQ3*, and *TRPC2*	6.0E-5
metal ion transmembrane transporter activity	*CACNB2*, *FGF2*, *KCNH1*, *KCNH7*, *KCNH8*, *KCNIP4*, *KCNK9*, *KCNMA1*, *KCNQ3*, and *TRPC2*	2.1E-4
voltage-gated ion channel activity	*CACNB2*, *FGF2*, *KCNH1*, *KCNH7*, *KCNH8*, *KCNIP4*, *KCNMA1*, and *KCNQ3*	2.6E-4
voltage-gated channel activity	*CACNB2*, *FGF2*, *KCNH1*, *KCNH7*, *KCNH8*, *KCNIP4*, *KCNMA1*, and *KCNQ3*	2.6E-4
potassium channel activity	*KCNH1*, *KCNH7*, *KCNH8*, *KCNIP4*, *KCNK9*, *KCNMA1*, and *KCNQ3*	4.2E-4
voltage-gated cation channel activity	*KCNH1*, *KCNH7*, *KCNH8*, *KCNIP4*, *KCNK9*, *KCNMA1*, and *KCNQ3*	4.9E-4
potassium ion transport	*KCNH1*, *KCNH7*, *KCNH8*, *KCNIP4*, *KCNK9*, *KCNMA1*, and *KCNQ3*	7.1E-4

1 P-value <0.0001.

Ion channel activity was associated with maintenance of normal gradient on plasma membranes, participation in cellular de- and re-polarization, neurotransmitter release, immune function, insulin secretion, and active transport mechanisms required for the digestion and absorption of nutrients [67], [68], [69].

**Figure 1:**
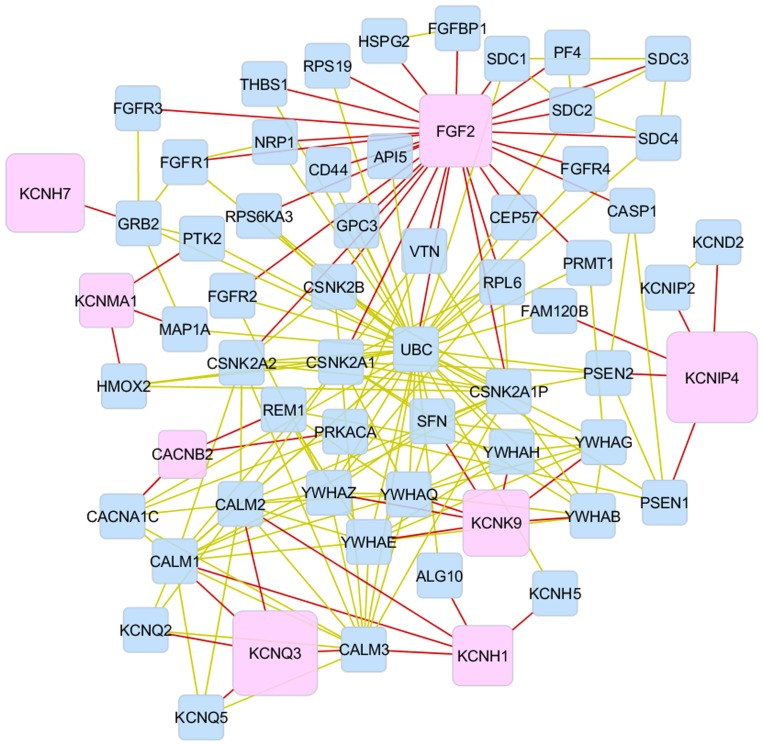
Network of genes affiliated to enriched molecular functions based on SNPs associated with feed efficiency. Connection between genes affiliated with enriched (P-value <0.0001) functional categories. Genes corresponding to detected (target genes; P-value <0.01) are represented by pink nodes, and intermediate (not-detected) genes are represented by blue nodes. Red edges represent in direct interaction with target genes with any other gene, and golden edges represent interactions between intermediate genes. The size of the network nodes from the target genes is a function of the P-values from the association analyses, in which larger nodes indicate more significant P-values.

A comprehensive network of the genes affiliated to the enriched molecular functions is reconstructed (Figure 1). In this network, the highest numbers of connections were displayed by the target gene *FGF2* and the intermediate gene ubiquitin C (*UBC*). These genes could have driver or hub role on feed efficiency components. In addition, *FGF2* was implicated on smooth muscle cell differentiation and signaling [31], [70].

## Conclusions

Single nucleotide polymorphisms associated with the feed efficiency components ADG and DMI in feedlot beef steers were identified using uni-SNP and multi-SNP models and univariate and bivariate analyses. The complementary set of SNPs detected by the univariate and bivariate analyses confirmed the value of considering both GWAS approaches. For certain SNPs, the bivariate analyses could gain precision through the consideration of covariation between ADG and DMI relative to univariate analyses. For other SNPs, univariate analyses could benefit from lower noise of each trait studied separately, relative to the bivariate analysis. Genomic loci that had favorable associations with ADG and DMI simultaneously, or favorable associations with either trait with minimum detrimental association with the other trait, while accounting for the body maintenance requirements, were identified. The validation of models and SNPs suggest that the findings could be replicable. Functional analysis and gene network visualization facilitated the interpretation of the association between SNPs mapping to genes that have ion channel-related molecular function and feed efficiency components. Results from this study can be used for genome-enabled improvement of feed efficiency in feedlot beef cattle, to support further empirical confirmation of the associations, and as proof of concept of the value of complementary association analyses.
